# Methylation Analysis of DNA Mismatch Repair Genes Using DNA Derived from the Peripheral Blood of Patients with Endometrial Cancer: Epimutation in Endometrial Carcinogenesis

**DOI:** 10.3390/genes7100086

**Published:** 2016-10-14

**Authors:** Takashi Takeda, Kouji Banno, Megumi Yanokura, Masataka Adachi, Moito Iijima, Haruko Kunitomi, Kanako Nakamura, Miho Iida, Yuya Nogami, Kiyoko Umene, Kenta Masuda, Yusuke Kobayashi, Wataru Yamagami, Akira Hirasawa, Eiichiro Tominaga, Nobuyuki Susumu, Daisuke Aoki

**Affiliations:** Department of Obstetrics and Gynecology, Keio University School of Medicine, Tokyo 160-8582, Japan; double.tak777@gmail.com or double.tak@keio.jp (T.T.); meguyano@keio.jp (M.Y.); madachi1014@keio.jp (M.A.); moito.not.for.sale@gmail.com (M.I.); harukoirie1225@gmail.com (H.K.); konakaramukana0101@hotmail.co.jp (K.N.); mihoiida1029@gmail.com (M.I.); yuya22wing@hotmail.com (Y.N.); umekiyo@hotmail.co.jp (K.U.); masuken1106@gmail.com (K.M.); kobax@a2.keio.jp (Y.K.); gami@z8.keio.jp (W.Y.); hir-aki@z8.keio.jp (A.H.); tominaga@a3.keio.jp (E.T.); susumu35@a6.keio.jp (N.S.); aoki@z7.keio.jp (D.A.)

**Keywords:** Lynch syndrome, epimutation, endometrial cancer, mismatch repair genes, methylation

## Abstract

Germline mutation of DNA mismatch repair (MMR) genes is a cause of Lynch syndrome. Methylation of MutL homolog 1 (*MLH1*) and MutS homolog 2 (*MSH2*) has been detected in peripheral blood cells of patients with colorectal cancer. This methylation is referred to as epimutation. Methylation of these genes has not been studied in an unselected series of endometrial cancer cases. Therefore, we examined methylation of *MLH1*, *MSH2*, and *MSH6* promoter regions of peripheral blood cells in 206 patients with endometrial cancer using a methylation-specific polymerase chain reaction (MSP). Germline mutation of MMR genes, microsatellite instability (MSI), and immunohistochemistry (IHC) were also analyzed in each case with epimutation. *MLH1* epimutation was detected in a single patient out of a total of 206 (0.49%)—1 out of 58 (1.72%) with an onset age of less than 50 years. The patient with *MLH1* epimutation showed high level MSI (MSI-H), loss of *MLH1* expression and had developed endometrial cancer at 46 years old, complicated with colorectal cancer. No case had epimutation of *MSH2* or *MSH6*. The *MLH1* epimutation detected in a patient with endometrial cancer may be a cause of endometrial carcinogenesis. This result indicates that it is important to check epimutation in patients with endometrial cancer without a germline mutation of MMR genes.

## 1. Introduction

Lynch syndrome is an autosomal dominant syndrome characterized by early development of colorectal and endometrial cancers, with an elevated risk of ovarian and other cancers [1]. Lynch syndrome accounts for 2%–6% of cases of endometrial cancer and is caused by germline mutations in DNA mismatch repair (MMR) genes (*MLH1*, *MSH2*, *MSH6*, and *PMS2*) [2]. Genomic rearrangements in the epithelial cell adhesion molecule (*EPCAM*) gene can also lead to a silencing of the closely linked *MSH2* gene and cause Lynch-associated cancers. These genes encode components of the mismatch repair system, which comprises two main protein complexes: the MutL homologue (MLH1, PMS2) and the MutS homologue (MSH2, MSH6). An aberration in one of these genes, and loss of these proteins, prevent appropriate repair of base mismatches produced during DNA replication.

Recent studies have shown that epigenetic changes play a significant role in carcinogenesis in various cancers, including endometrial cancer [3]. In particular, the relationship between hypermethylation of specific genome regions and carcinogenesis has attracted attention [4,5,6,7,8]. Errors in epigenetic gene repression, including methylation, are referred to as epimutation. Epimutation in MMR genes, especially in *MLH1* and *MSH2*, has been reported in colorectal cancers, and these cases show an early-onset Lynch syndrome-like phenotype [9,10,11,12,13,14,15,16,17,18,19]. *MSH6* and *PMS2* epimutation has never been reported. Although about one-third of females with an *MLH1* epimutation developed endometrial cancer, and one case with an *EPCAM* deletion linked to *MSH2* epimutation also developed endometrial cancer [12,20], constitutional methylation of the MMR genes has not previously been studied in an unselected series of endometrial cancer cases. Here, we report the first investigation of epimutation in MMR genes using peripheral blood DNA samples from unselected patients with endometrial cancer.

## 2. Materials and Methods

### 2.1. Subjects

The subjects were 206 consecutive patients with endometrial cancer who visited our hospital for treatment or follow-up from January 2012 to March 2015. All patients gave written informed consent, and the study was approved by the institutional review board (No. 2013–0258, No. 2007–0081). Peripheral blood was collected from all patients, and DNA was extracted from lymphocytes. Methylation of the promoter regions of MMR genes (*MLH1*, *MSH2*, and *MSH6*) was analyzed by a methylation-specific polymerase chain reaction (MSP). Methylation analysis by MSP was performed twice for each sample. Age at onset, pathological type, International Federation of Gynecology and Obstetrics (FIGO) stage, history, and family history were collected from medical records and an interview. Patients’ characteristics are shown in Table 1. Six patients had a history of other cancers (rectum: 2, breast: 3, ovary: 1), and 27 had a family history of cancer in a first-degree relative (including rectum: 12, stomach: 8, breast: 4). Six cases fulfilled the revised Bethesda guidelines. DNA sequencing to detect germline mutation of MMR genes, microsatellite instability (MSI) tests in cancer regions to detect loss of function in MMR, and immunohistochemistry (IHC) to visualize expression of MMR proteins were also performed in cases identified with constitutional methylation of an MMR gene or in cases where these tests were otherwise clinically indicated (*n* = 24).

### 2.2. Methylation Analysis

The methylation status of CpG islands in the *MLH1*, *MSH2*, and *MSH6* promoters was analyzed by MSP. This method detects methylation of bases by a bisulfite reaction and subsequent detection by polymerase chain reaction (PCR) with primers specific to bisulfite converted methylated and unmethylated sequences. We referred to the primers and PCR conditions for MSP analysis as previously reported [21,22]. MSP analyses for *MLH1*, *MSH2*, and *MSH6* are summarized in Table 2. CpGenome Universal Methylated DNA and Unmethylated DNA (Millipore, Temecula, CA, USA) were used as the respective positive controls for methylated and unmethylated PCR. The MSP products were subsequently sequenced to confirm the presence of methylation in the methylated MSP.

### 2.3. Germline Mutation Analysis

Mutation screening of MMR genes was performed by direct sequencing, as previously reported [4,23].

### 2.4. Microsatellite Instability Analysis

To detect the loss of function in MMR, five satellite markers (BAT25, BAT26, D2S123, D5S346, and D17S250) were evaluated, as recommended by the National Cancer Institute (NCI) [24]. MSI analysis was performed as previous reported [4]. PCR primers for these regions were purchased from Research Genetics (Huntsville, AL, USA). The antisense primers contained a fluorescent marker, Cy5 amidite (indodicarbocyanine), at the 5′ end. AmpliTaq polymerase and AmpliTaq buffer (Perkin-Elmer, Boston, MA, USA) were used in the PCR, in which 1 μL of sample DNA (template, 0.1 μg/μL) was added to 24 μL of premixture (distilled water, 16.125 μL; 1.25 mmol/L deoxyribonucleotide triphosphate (dNTP), 4 μL; 10× BPCR buffer, 2.5 μl; optical density (OD) 2.2 forward primer, 0.625 μL; OD 2.2 reverse primer, 0.625 μL; and Taq polymerase, 0.125 μL) in a total reaction volume of 25 μL. Forward and reverse primers specific for D2S123, D5S376, D17S250, BAT25, and BAT26 regions were used. DNA denaturation at 95 °C for 5 min was followed by 40 cycles for 30 s at 95 °C, 40 s at 55 °C, and 40 s at 72 °C. The reaction mixture was then heated to 72 °C for 7 min, cooled, and stored at 4 °C. PCR products were combined with size markers, denatured for 5 min at 80 °C, and then electrophoresed in a 6% Long Ranger 7 M urea denaturing gel on an AFL red DNA sequencer (Amersham Pharmacia Biotech, Tokyo, Japan). DNA fragment sizes were analyzed using gene scanning software (Allele Links; Amersham Pharmacia Biotech). Tumors showing an allelic shift of two or more markers were referred to as high level MSI (MSI-H), those with an allelic shift of one marker as low level MSI (MSI-L), and those with no allelic shift at any marker as microsatellite stable (MSS).

### 2.5. Immunohistochemistry

Immunohistochemical analysis was performed as previously described [25]. Tissue was fixed overnight with 4% paraformaldehyde, embedded in paraffin, and prepared as sections of 4 µm. Sections depleted of paraffin were immunostained with primary antibodies against MLH1 (551092; BD, Franklin Lakes, NJ, USA), MSH2 (sc-494; Santa Cruz Biotechnology, Santa Cruz, CA, USA), and MSH6 (610919; BD), followed by peroxidase-conjugated secondary antibodies (ENVISION+; Dako, Glostrup, Denmark). Sections were counterstained with hematoxylin to visualize cell nuclei. The staining results were independently evaluated by two trained investigators.

## 3. Results

**Figure 1:**
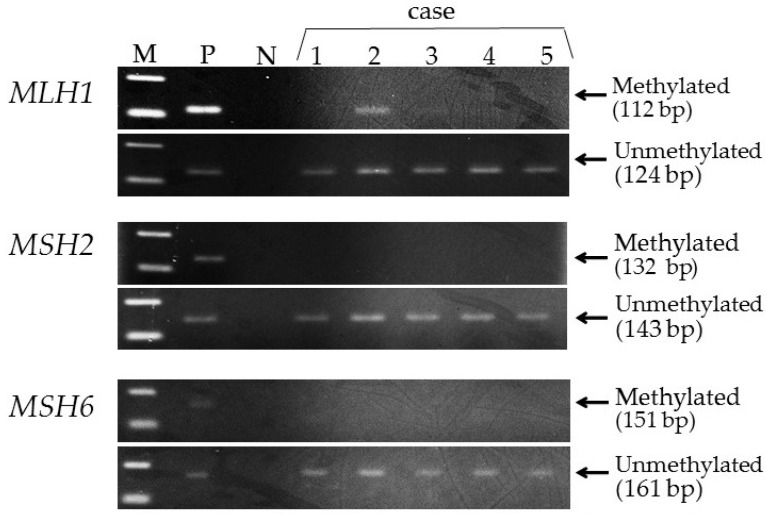
Methylation specific polymerase chain reaction (MSP) using peripheral blood lymphocytes. The patient (case 2) had methylation of *MLH1*, but not of *MSH2* and *MSH6*. M: marker; P: positive control; N: negative control.

**Figure 2:**
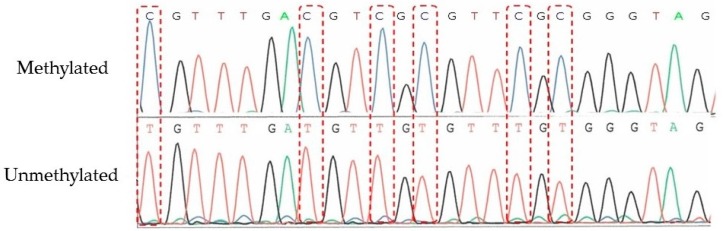
DNA sequencing of the *MLH1* promoter region after a bisulfite reaction in the case with epimutation. Methylated bases were not converted after bisulfite reaction and epimutation of *MLH1* was confirmed.

**Figure 3:**
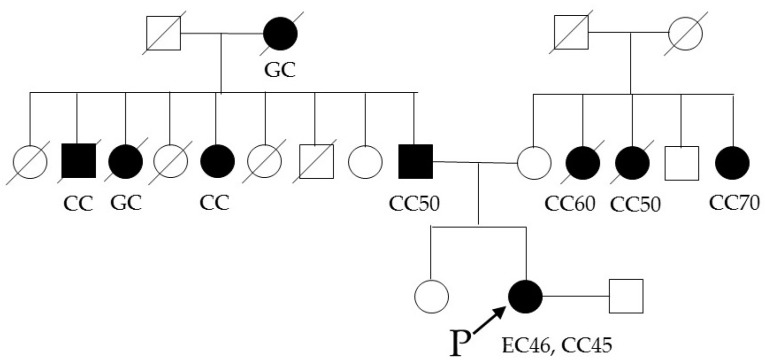
Family tree of the patient with *MLH1* epimutation. The patient had familial accumulation of Lynch-associated cancers, met the revised Bethesda guidelines, and met the Amsterdam II criteria. Numbers written in the figure are age at onset when known. CC: colorectal cancer, EC: endometrial cancer, GC: gastric cancer.

**Figure 4:**
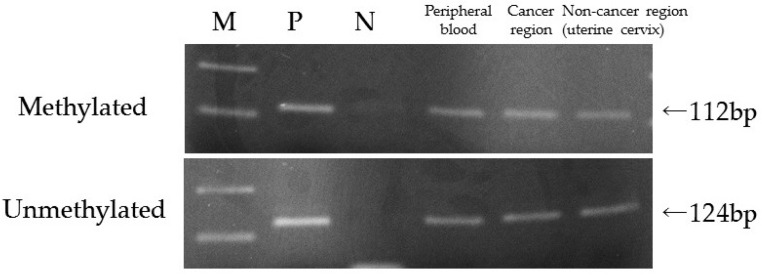
Additional MSP using uterine cervix tissue as a non-cancer region. All DNA is from peripheral blood lymphocytes; the cancer region and the non-cancerous uterine cervix showed *MLH1* methylation in this case. M: marker; P: positive control; N: negative control.

**Figure 5:**
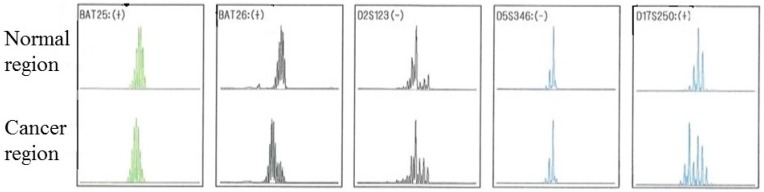
Checking microsatellite instability (MSI) with five satellite markers, as recommended by National Cancer Institute (NCI). BAT25, BAT26, and D17S250 were positive, showing high level MSI (MSI-H).

**Figure 6:**
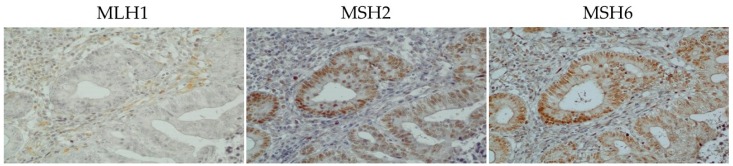
Immunohistochemistry for MMR proteins. MLH1 was absent (negative staining). MSH2 and MSH6 showed normal positive staining.

One of the 206 patients (0.49%) was found to have *MLH1* epimutation. Figure 1 shows the MSP of this patient and the *MLH1* methylation in peripheral blood lymphocytes. Direct sequencing of methylated and unmethylated MSP products from blood lymphocytes confirmed methylation in the *MLH1* promoter region in this case (Figure 2). This patient had endometrial cancer at 46 years old: endometrioid adenocarcinoma G1 with post-surgical stage IA (FIGO 2008). She had a history of colorectal cancer at 45 years old and met the revised Bethesda guidelines because of the early onset of colorectal cancer and metachronous Lynch-associated cancers (Table 3). Based on the familial clustering of Lynch syndrome-associated cancers and the age of onset, this family met the Amsterdam II criteria for Lynch syndrome. We could not definitively rule out the possibility of familial adenomatous polyposis (FAP) because the medical records of her colorectal cancer and her relatives’ cancers were unavailable to us, since they were treated at other hospitals. Nevertheless, FAP seems unlikely, given the proband was not advised of any diagnosis of polyposis following her surgery for colorectal cancer. Germline genetic testing showed no mutation of MMR genes (*MLH1*, *MSH2*, and *MSH6*). Her family tree is shown in Figure 3. Methylation was also found in uterine cervix tissue (non-cancer region) and in a peripheral blood sample, while the cancerous region of the uterine endometrium had methylated and unmethylated alleles (Figure 4). MSI tests with five satellite markers showed MSI-H, which indicated the possibility of deficiency in MMR (Figure 5). IHC of MMR proteins (Figure 6) clearly showed decreased MLH1 expression in the adenocarcinoma region. As indicated above, this case met the Amsterdam II criteria and six cases fulfilled the revised Bethesda guidelines. In patients under 50 years old, the rate of epimutation was 1 in 58 patients (1.72%). Twenty-four patients, including this epimutation case, in 206 patients were clinically recommended to be checked for MSI; seven patients showed MSI, and another 17 patients showed MSS.

## 4. Discussion

This is the first study on the relationship of the epimutation of MMR genes in unselected endometrial cancer cases. Epimutation was first reported in 2002 in a case of colorectal cancer by Gazzoli et al., based on methylation in a single allele of the *MLH1* promoter in peripheral blood DNA [9]. Multiple cases of epimutation in MMR genes have subsequently been described [10,11,12,13,14,15,16,17,18,19]. Based on these cases, the NCCN Clinical Practice Guidelines in Oncology (NCCN Guidelines) suggest additional tests for epimutation in some cases of colorectal cancers [26]. However, the best approach for endometrial cancer is unclear. Our results suggest that the epimutation of MMR genes should be considered in the evaluation of cases of endometrial cancers.

**Figure 7:**
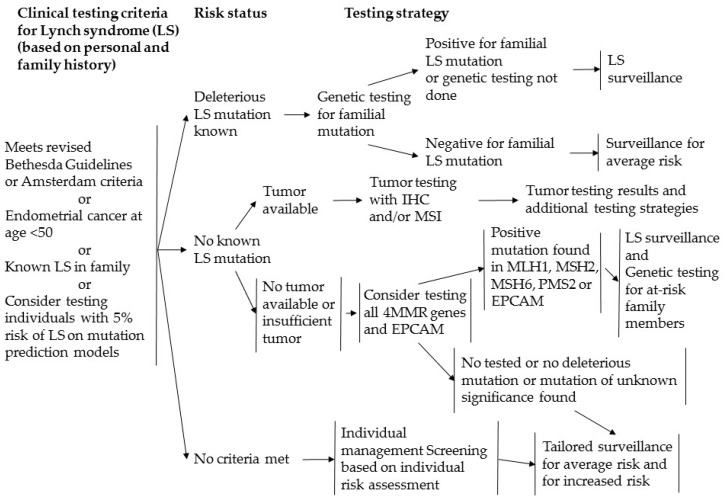
Screening for Lynch syndrome in the NCCN guidelines. EPCAM: epithelial cell adhesion molecule; IHC: immunohistochemistry.

**Figure 8:**
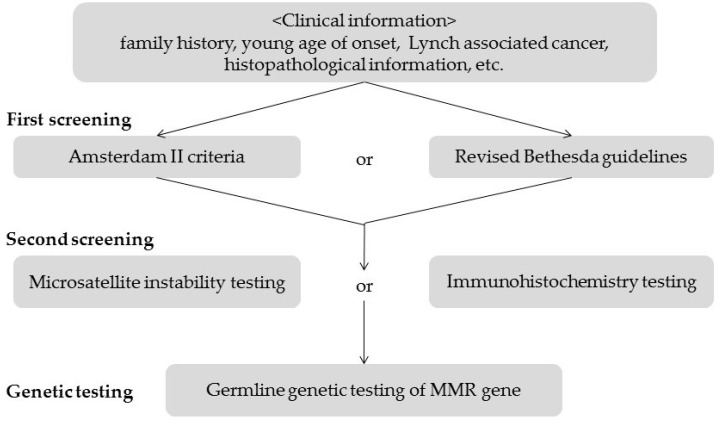
Screening for Lynch syndrome in the Japanese Clinical Practice Guidelines for Hereditary Colorectal Cancer.

Screening and testing for Lynch syndrome in the NCCN Guidelines and the Japanese Clinical Practice Guidelines for Hereditary Colorectal Cancer are shown in Figure 7 and Figure 8 [26,27]. MSI tests, or IHC, are recommended for patients with clinical information on family history, young age at onset, and Lynch-associated cancer, and patients who fulfill the Amsterdam II criteria or the revised Bethesda guidelines. In cases with MSI-H or negative staining for one or more MMR proteins, germline genetic tests should then be considered. The NCCN guidelines indicate that patients with endometrial cancer at ages less than 50 years old should be considered for testing for Lynch syndrome. In the current study, the patient with *MLH1* epimutation was 46 years old, had a history of colorectal cancer (Lynch-associated cancer), had a family history of Lynch-associated cancers, and met the revised Bethesda guidelines. Additionally, she had MSI-H and IHC showed a loss of expression of MLH1. The screening strategy recommends germline genetic testing of MMR genes; however, an epimutation results in normal DNA sequencing and cannot be revealed without the use of a methylation detection technique, e.g., MSP.

Our case may explain why some patients do not have germline mutations in MMR genes, despite fulfilling the Amsterdam II criteria or the revised Bethesda guidelines, and having MSI or loss of MMR proteins. Mangold et al. found germline mutations of *MLH1* or *MSH2* in 62% of patients who fulfilled the revised Bethesda guidelines and had MSI-H [28]. Colorectal cancer patients with suspected Lynch syndrome, who were selected for epimutation tests because of negative germline tests for MMR genes, had MSI and IHC loss of MLH1 due to epimutation at rates of 3%–10% [11,13,14,16,17,29,30,31,32,33]. The current study suggests that similar findings may occur in endometrial cancer. MSI or IHC were not performed for all patients because of the limitation of the setting of this study and the unavailability of cancer tissue. In 24 of 206 patients, MSI was checked, as clinically recommended; seven patients showed MSI, and another 17 patients showed MSS. *MLH1* epimutation was detected only from these seven patients. Further research for detecting the epimutation of MMR genes in selected groups (e.g., the MSI-H group, IHC loss of the MMR group, or both) is needed. These previous reports and our findings may support the routine evaluation of the epimutation of MMR genes in cases of suspected Lynch syndrome fulfilling diagnostic criteria, showing MSI and lacking expression of MMR proteins.

We also checked *MSH2* and *MSH6* for epimutation. No case of *MSH6* epimutation has ever been described, and also there was no *MSH6* epimutation in our study. No *MSH2* epimutation was detected in our study either; however, methylation screening of *MSH2* is no longer an appropriate analysis for detecting *MSH2* epimutation. This is because *MSH2* epimutation is a secondary consequence of germline deletion of the *EPCAM* gene, and the standard test is multiplex ligation probe amplification (MLPA) analysis to detect deletion of the 3′ end terminal of the *EPCAM* gene [34]. Additionally, even among cases with an *EPCAM* deletion, hypermethylation and silencing of *MSH2* is restricted to cells expressing EPCAM. This means *MSH2* epimutation caused by *EPCAM* deletion is not always detected in blood cells because they are not epithelial cells. Additionally, carriers of *EPCAM* deletions show mosaic patterns of *MSH2* inactivation, and these features may lead to false-negative results. While expression levels of EPCAM vary in the tissue, *EPCAM* deletion with consequent *MSH2* epimutation has a low risk for endometrial cancer development against a high risk for colorectal cancer [35]. We could not check for *EPCAM* mutation and MLPA analysis for *MSH2*, and further investigation is needed.

Studies of mature spermatozoa from male *MLH1* epimutation carriers have shown that *MLH1* methylation is absent in spermatozoa, while methylation is present soma-wide [13,31,36,37]. Constitutional epimutation is the term describing the epigenetic change found in somatic tissues, but is absent or erased in the germline. Additionally, it is identified by finding hemiallelic methylation of the promoter lesion in all three embryonic germ cell lineages: endodermal (buccal and colonic epithelium), mesodermal (blood lymphocytes), and ectodermal (hair follicles) [38,39,40]. In the current study, we found the *MLH1* methylation in blood lymphocytes and uterine cervix for normal tissue; however, they are both mesodermal and it would be better to check for methylation in ectoderm and endoderm to clearly determine *MLH1* epimutation. Furthermore, validation of the presence and level of methylation should be performed using at least two independent methods, such as bisulfite pyrosequencing and clonal bisulfite sequencing. We used MSP to detect methylation for screening and confirmed with bisulfite sequencing; however, to clarify whether the epimutation occurred in hemiallelic or mosaic forms, bisulfite pyrosequencing should also be performed for detecting the ratio of methylation. Although most *MLH1* epimutation are characterized by monoallelic methylation, in this case, it would be expected to be due to monoallelic methylation [13,16,41].

Most cases identified with a constitutional *MLH1* epimutation have a personal history of Lynch syndrome-like tumors, but no family history. This is because *MLH1* epimutation tends to arise de novo on the maternal allele [11,13,15,31,42]. Detecting epimutation of *MLH1* may be useful for patients themselves, and for their families. The lifetime risk of cancers in patients with epimutation of MMR genes has not been examined, in contrast to Lynch syndrome. However, since *MLH1* epimutation has the same effects as *MLH1* germline mutation for the patients themselves, following patients with epimutation of MMR genes may be helpful for surveillance and early detection of Lynch-associated cancers. Additionally, some patients and their first-degree relatives who are suspected for Lynch syndrome with an uninformative genetic test result are needed to undergo clinical surveillance. As such, detecting epimutation of *MLH1* of patients can prevent the unnecessary clinical surveillance for their families because of a low incidence of inheritance of *MLH1* epimutation. In this study, the patient with *MLH1* epimutation almost met the Amsterdam II criteria; she clearly had familial accumulation, indicating the possibility of inheritable constitutional epimutation. We were unable to collect DNA samples from the patient’s family members; therefore, we do not know whether this epimutation was inheritable or not. This autosomal dominant transmission of *MLH1* epimutation was recently described as a secondary *MLH1* epimutation. This inheritable secondary *MLH1* epimutation is thought to link to a germline promoter c.-27C>A or c.85G>T single nucleotide variant (SNV), or both, in European ancestry, as previously reported [36,43]. This epimutation was erased in the spermatozoa, the same as the constitutional epimutation, even though the epimutation will be apparent soma-wide in the offspring. This indicated that the *MLH1* epimutation is erased in the germline and reset in each member of the next generation who inherits the c.-27C>A or c.85G>T genetic alleles, or both. It is possible the proband described here also has an underlying genetic alteration causing the epimutation, given her family history of cancer. Although sequencing of the *MLH1* gene did not identify any coding mutation, it remains possible that another genetic alteration around *MLH1* exists in this family, but this was not investigated in this study due to the lack of available samples and consent.

The novelty of this study is that the subjects were unselected patients with endometrial cancer, and epimutation has not previously been reported on a patient series ascertained on the basis of the development of endometrial cancer. However, a limitation of this study was that the cancers from this patient series were not systematically screened for MSI, according to universal testing schemes for Lynch syndrome. Therefore, we are unable to infer the frequency of *MLH1* epimutation among cases whose cancer exhibited MSI. In patients with colorectal cancer, Castillejo et al. [17] found *MLH1* constitutional epimutation in five of 847 patients who met the revised Bethesda guidelines (0.59%), but in zero of 2123 patients who did not meet the guidelines. For comparison, *MLH1* germline mutation was detected in 18 of the cases that met the guidelines (2.13%). Among 106 patients who met the guidelines and showed a loss of MLH1 on IHC, five had *MLH1* constitutional epimutation (4.7%) [17]. This result indicates that constitutional *MLH1* epimutation may be found more frequently in a subgroup showing MSI-H or IHC loss of MMR, meeting the guidelines with endometrial cancer, or all three. Further cases and subgroup analysis are required.

## 5. Conclusions

*MLH1* epimutation was detected in a single patient out of 206 (0.49%) with endometrial cancer. This finding indicates that the epimutation of MMR genes should be evaluated in cases of suspected Lynch syndrome that fulfill the revised Bethesda guidelines, show MSI, and lack expression of MMR proteins.

## Figures and Tables

**Table 1:** Clinical characteristics of the patients (*n* = 206).

Histological Type		Number of Patients	%	Mean Age		
Endometrioid	G1	105	51.0	54.9 (43.3–66.5)		
	G2	54	26.2	Surgical stage *	Number of patients	%
	G3	23	11.1	I	155	75.2
Serous		13	6.3	II	16	7.8
Clear		5	2.4	III	29	1.4
Adenosquamous		3	1.5	IV	6	2.9
Undifferentiated		3	1.5	* (FIGO 2008)		

FIGO: International Federation of Gynecology and Obstetrics.

**Table 2:** Primer sequences used in methylation-specific polymerase chain reaction (MSP).

Gene Name	PCR Analysis	Sense	Antisense	Size (bp)	Annealing Temp (°C)
*MLH1*	Methylated	ACGTAGACGTTTTATTAGGGTCGC	CCTCATCGTAACTACCCGCG	112	60
	Unmethylated	TTTTGATGTAGATGTTTTATTAGGGTTGT	ACCACCTCATCATAACTACCCACA	124	60
*MSH2*	Methylated	TCGTGGTCGGACGTCGTTC	CAACGTCTCCTTCGACTACACCG	132	66
	Unmethylated	GGTTGTTGTGGTTGGATGTTGTTT	CAACTACAACATCTCCTTCAACTACACCA	143	66
*MSH6*	Methylated	TTTTTTCGGCGGAGCGC	AAAAAAAAACTATACAAAATACTCTATCGC	151	62
	Unmethylated	TTTGGGTTTTTTTGGTGGAGTGT	CTTAAAAAAAAAACTATACAAAATACTCTATCACA	161	62

**Table 3:** Amsterdam II Criteria and revised Bethesda guidelines.

**Amsterdam II Criteria**
There should be at least three relatives with a hereditary nonpolyposis colorectal cancer (HNPCC)-associated cancer
[colorectum, endometrium, small bowl, ureter, renal pelvis]
All of the following criteria should be present:
1. One should be a first-degree relative of the other two.
2. At least two successive generations should be affected.
3. At least one should be diagnosed at <50 years of age.
4. FAP should be excluded in the colorectal cancer cases, if any.
5. Tumors should be verified by pathological examination.
**Revised Bethesda Guidelines**
Tumors from individuals should be tested for MSI in the following situations:
1. Colorectal cancer diagnosed in a patient who is less than 50 years of age.
2. Presence of synchronous, metachronous colorectal, or other Lynch-associated tumors * regardless of age.
3. Colorectal cancer with microsatellite instability-high (MSI-H) histology ** diagnosed in a patient who is less than 60 years of age.
4. Colorectal cancer diagnosed in one or more first-degree relatives with a Lynch-related tumor, with one of the cancers being diagnosed under age 50 years.
5. Colorectal cancer diagnosed in two or more first- or second-degree relatives with Lynch-related tumors, regardless of age.

Abbreviations: MSI: microsatellite instability; MSI-H: high-frequency microsatellite instability. FAP: familial adenomatous polyposis. * Lynch syndrome–related tumors include colorectal, endometrial, stomach, ovarian, pancreatic, ureter and renal pelvic, biliary tract, and brain (usually glioblastoma, as seen in Turcot syndrome) tumors; sebaceous gland adenomas and keratoacanthomas in Muir-Torre syndrome, and carcinomas of the small bowel. ** Presence of tumor-infiltrating lymphocytes, Crohn’s-like lymphocytic reaction, mucinous/signet-ring differentiation, or medullary growth pattern.
